# cAMP Responsive Element Binding Protein-1 Is a Transcription Factor of Lysosomal-Associated Protein Transmembrane-4 beta in Human Breast Cancer Cells

**DOI:** 10.1371/journal.pone.0057520

**Published:** 2013-02-28

**Authors:** Meng Zhang, Jian-Jun Xu, Rou-Li Zhou, Qing-Yun Zhang

**Affiliations:** 1 Department of Clinical laboratory, Key laboratory of Carcinogenesis and Translational Research (Ministry of Education), Peking University Cancer Hospital & Institute, Beijing, China; 2 Department of Cell Biology, School of Basic Medical Sciences, Peking University, Beijing, China; University of Saarland Medical School, Germany

## Abstract

Lysosomal-associated protein transmembrane-4 beta (LAPTM4B) is a potential proto-oncogene, whose overexpression is involved in cancer occurrence and progression. Its transcript is up-regulated in various types of solid tumors including breast cancer. However, its transcriptional regulation mechanism is still unclear. To investigate the mechanism of transcriptional regulation of LAPTM4B in human breast cancer cells, a series of luciferase reporter constructs and construct with mutated binding site for cAMP responsive element binding protein-1 (CREB1) were generated by PCR amplification and transiently transfected into breast cancer cells to determine the transcriptional activities of different promoter regions. The +10∼+292 promoter region was possessed the highest transcriptional activity. The ability of CREB1 to bind the LAPMT4B promoter was confirmed by electrophoretic mobility shift assay, super-shift and RNA interference experiments. Our study identified the core promoter region responsible for constitutive expression of LAPTM4B and clarified that CREB1 played an important role in LAPTM4B transcriptional regulation in human breast cancer cells.

## Introduction

Lysosomal-associated protein transmembrane-4 beta (LAPTM4B) was first cloned in hepatocellular carcinoma (HCC), encoding a type III transmembrane protein with four transmembrane regions. The localization of LAPTM4B was found not only to Lysosome, but also to the plasma membrane and internal organelles such as Golgi and Endosomes [1]. It has been reported that LAPTM4B was widely expressed in normal human tissues and obviously up-regulated in various types of carcinomas [2]. The overexpression of LAPTM4B is associated with unfavorable biological behaviors and poor prognosis of many carcinomas, such as breast cancer [2], HCC [3]; [4]; [5], gallbladder carcinoma [6]; [7], colorectal carcinoma [8], epithelial ovarian carcinoma [9], endometrial carcinoma [10] etc. LAPTM4B is able to bind to multidrug resistance 1 (MDR1), and also active PI3K/AKT signaling pathway, which motivates multi-drug resistance [11]. Recent studies showed that the amplification of LAPTM4B was associated with breast cancer recurrence and chemotherapy resistance [12]. Northern blots revealed an overexpression of LAPTM4b mRNA in breast cancer [2]. The overexpression of LAPTM4B was considered as being caused by gene amplification [12] and transcriptional up-regulation [13]. However, the specific transcription factors of LAPTM4B remain unclear.

cAMP responsive element binding protein-1 (CREB1) is a transcription factor that plays an important role in cell proliferation, differentiation, and survival [14]; [15]. The protein is a member of the leucine zipper family of DNA binding proteins. It has been reported that CREB1 regulated the expression of many genes in response to hormones, ion fluxes, growth factors, and stress signals. cAMP responsive element (CRE) is the CREB1 binding site. It not only lay near 5′ ends, but also relative to introns, exons and transcriptionally active regions (TARs) of target genes [16]. Many studies showed that CREB1 was related to neuropsychiatric diseases such as neurodegeneration [17] and Alzheimer disease [18]. CREB1 was also regarded as an oncogene that promoted tumor cell growth and proliferation [19]. Identified downstream genes of CREB1 include the oncogene bcl-2, the cell cycle-related genes cyclinA1, cyclinB1, and cyclinD2, signal transduction proteins, activated transcription factor 3, NF-κB, and other growth-related genes. Recent studies also showed that CREB1 played a key role in estrogen receptor-negative breast cancer [20]; [21].

In this study, we analyzed the LAPTM4B promoter region and the first exon. A CREB1 conserved binding region in the first exon was found. The function of CREB1 on LAPTM4B transcriptional regulation was explored by electrophoretic mobility shift assay (EMSA), super-shift and luciferase reporter experiments. The expression of CREB1 was knocked down by RNA interference, and we also observed the synchronous decline of LAPTM4B mRNA. Explicit mechanism of LAPTM4B transcriptional regulation will help us explicate the overexpression of LAPTM4b mRNA in breast cancer and understand the relationship between LAPTM4B gene and other proteins.

## Materials and Methods

### Computer Analysis of the Promoter Region

The 5′-genomic sequences of LAPTM4B of different species were gained from GenBank. Promoter prediction and potential transcription factor binding site analysis was performed using the IFTI-Mirage and JASPAR website online programs (http://www.ifti.org and http://jaspar.genereg.net/cgi-bin/jaspar_db.pl). The nucleotide sequences are numbered with the transcription start site as +1. A 501 bp (−200 ∼ +301) Genomic DNA fragment was predicted as LAPTM4B promoter region. Profile score threshold was set to 95%.

### Cells and Culture Conditions

Human breast cancer cells: MCF7, T-47D, ZR-75-1 were maintained in Dulbecco’s modified Eagle’s medium (DMEM; GIBCO) supplemented with 10% fetal bovine serum (MIN HAI BIO-ENGINEERING) and antibiotics. Cells were grown under standard cell-culture conditions. All the breast cancer cells were kindly provided by Dr. SHOU Cheng-chao of Department of Biochemistry and Molecular Biology, Peking University Cancer Hospital & Institute. Dr. SHOU obtained these three cell lines from the American Type Culture Collection (ATCC). The ATCC® Number for MCF7, T-47D and ZR-75-1 were HTB-22™, HTB-133™ and CRL-1500™ respectively.

### Promoter Construct

A 283 bp of the human LAPTM4B promoter region was amplified from genomic DNA with primers AAA CTC GAG CCC TTG AAT GGA GTT ACA CGA ACG (sense, Xho I cutting site underlined) and AAA AAG CTT CTG CCT CCT CGA TAC CCC GAG A (anti-sense, Hind III cutting site underlined), using Pfu DNA polymerase. Taq DNA polymerase was used for overhang A synthesis. The PCR products were ligated into pGM-T vector (TIANGEN). The recombinant pGM-T vector was delivered for sequencing and digested with Hind III-Xho I (Promega). Digested products were purified with gel midi purification kit (TIANGEN) and cloned into Hind III-Xho I -restricted pGL3-Basic vector to generate pGL3-+10/+292.

CREB1 binding site inside the LAPTM4B core promoter was site-directed mutated by SOE-PCR using modified reverse primer spanning the CREB1 binding site (sense: GGCTGGAGCCCGCGATG*TG*GTCACGGACTCGGGTCACATGG and CCATGTGACCCGAGTCCGTGAC*CA*CATCGCGGGCTCCAGCC anti-sense, italics depicted the mutated bases). The products were also delivered for sequencing and cloned into pGL3 vector to generate pGL3-mut-+10/+292. The other five truncated plasmids were conserved by our laboratory.

### Transient Transfection and Luciferase Reporter Assay

Breast cancer cells were planted into 24-well plates in 6×10^4^ per well, and incubated for 24 h in complete medium as described before. Then MCF7, T-47D or ZR-75-1 were transfected with 1 µg of either pGL3 reporter constructs with various LAPTM4B promoter inserts, pGL3-basic vector, or pGL3-promoter vector. A 0.02 µg phRL-CMV plasmid was additionally transfected in each well to normalize for transfection efficiency. Transfection was carried out using Lipofectamine 2000 transfection reagent (Invitrogen) according manufacturer instructions. At 48 h after transfection, cell lysates were collected for Firefly and Renilla luciferase activities detection using DualGlo Luciferase Assay System (Promega). All reactions were run in triplicate and results were normalized using Renilla luciferase activity. The luciferase value of pGL3-promoter (positive control) was set to 100% and the other constructs were compared with it.

### Preparation of Unclear Extracts

MCF7 cell were harvested, washed by PBS for twice, centrifugation by 1500 rpm for 5 min, and resuspended in Buffer A (10 mM HEPES,PH = 7.9, 1.5 mM MgCl_2_, 10 mM KCl, 0.5 mM DTT, 0.5 mM PMSF, 1×Protease Cocktail Inhibitor). After incubation on ice for 10 min, the nuclei were briefly vortexed, and centrifuged at 3000 rpm for 15 min. The precipitate was resuspended in Buffer B (20 mM HEPES, PH = 7.9, 0.2 mM EDTA, 1.5 mM MgCl_2_, 20 mM KCl, 25% glycerol, 0.5 mM DTT, 0.5 mM PMSF, 1×Protease Cocktail Inhibitor), and then Buffer C (20 mM HEPES, PH = 7.9, 0.2 mM EDTA, 1.5 mM MgCl_2_, 1.2 M KCl, 25% glycerol, 0.5 mM DTT, 0.5 mM PMSF, 1×Protease Cocktail Inhibitor) was added slowly. After incubation at 4°C for 30 min on a rotary shaker, the mixture was centrifuged at 14000 rpm for 30 min. The supernatant was used as nuclear extract. The protein concentration was determined using a bicinchoninic acid (BCA) protein assay kit (Applygen).

### EMSA and Super-shift Assays

The annealed double-strand oligonucleotide derived from the LAPTM4B promoter containing either the wild type (AGC CCG CGA TGA CGT CAC GGA CTC G) or mutated CREB1 binding site (AGC CCG CGA TGt gGT CAC GGA CTC G) were 3′ end-labeled by biotin using labeling kit (Pierce). The CREB1-binding site was underlined and the mutated nucleotides were denoted in lowercase.

A sample of nuclear protein (5 µg) was mixed for 20 min at room temperature with biotin-labeled oligonucleotide using an EMSA kit (Thermo Scientific) according manufacturer instructions. The mixture was subjected to 4% polyacrylamide gel electrophoresis (PAGE) for 60 min in 0.5% Tris-borate EDTA (TBE) buffer and transferred to a nylon transfer membrane (Hybond-N+) using the Trans-Blot SD semidry transfer cell system (Bio-Rad).The membrane was transferred to an ultraviolet (UV) cross-linker for 90s. The biotin end-labeled DNA was detected using the Streptavidin-Horseradish Peroxidase Conjugate and the Chemiluminescent Substrate. And the membranes were exposed to X-ray film (Kodak).

For the super-shift assay, 0.2 µg, 0.4 µg, and 0.8 µg of anti-CREB1 antibodies (Santa Cruz, C-21) were added to the binding reaction after 20 min of pre-incubation nuclear protein. Incubation was continued 20 min at room temperature.

### CREB1 mRNA Interference

MCF7 cells were transfected with 1 µg of shRNA plasmid (sc-29281-SH)(Santa Cruz Biotechnology Inc.) per well in 6-well plates, using shRNA plasmid transfection reagent (sc-108061)(Santa Cruz) according to the manufacturer instruction. Control shRNA plasmid-A (sc-108060) (Santa Cruz) was used as a negative control for shRNA transfection. 48 h after transfection cells were harvested for protein and RNA extraction.

### Western Blot Analysis

Cells were washed twice in ice-cold PBS and lysed using RIPA extraction reagent (TIANGEN).Whole cell lysates were boiled in lysis buffer containing 2% SDS. Protein concentration was determined by BCA protein assay kit (Applygen).The antibody used for western blot was anti-CREB1 (Santa Cruz, C-21). The intensity of gray value was measured by Image J 1.46 for Windows. For normalization of the target, β-actin was used as internal reference.

### Reverse Transcription and Real-time PCR Assay

Total RNA was isolated from breast cell lines using Trizol (TIANGEN) according to manufacturer instructions. Reverse transcription of 2 µg total RNA was carried out using oligo (dT16) primer. Quantitative real-time PCR was performed on a Roche 480 using SYBR Green Real-time PCR Master Mix (QPK-201) (TOYOBO). The primers for CREB1 were AAG ACC ATT AGA AAG CAC CAG G (forward) and ACC AAT CTA GGG CAG AAC ACT TA (reverse). Amplification of the GAPDH gene was used as an internal control for all real-time PCR reaction. The primers for glyceraldehyde-3-phosphate dehydrogenase (GAPDH) were ACG GAT TTG GTC GTA TTG GGC G (forward) and CTC CTG GAA GAT GGT GAT GG (reverse). All reactions were run in triplicate.

### Statistical Analysis

Biostatistical analysis was carried out using SPSS Statistics 16.0 for Windows. The normal distribution and the homogeneity test of variance were analyzed before the variance analysis. A one-way analysis of variance (ANOVA) was applied for the comparison of parametric data followed by the least significant difference (LSD) multiple range test to detect significant differences among the different groups. All error bars depict ±SD. The results were considered significant at *p*<0.05.

## Results

### Computer Analysis of the LAPTM4B Gene

**Figure 1:**
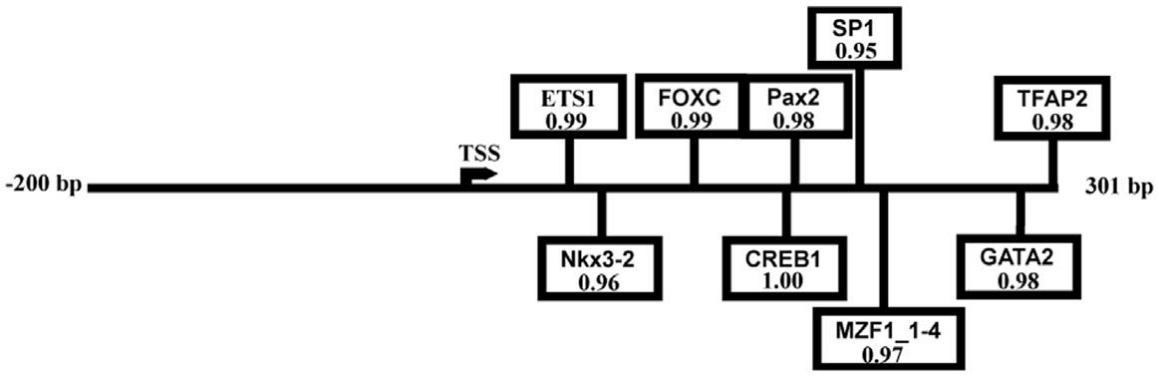
The schema of forecasting transcription factor in the human LAPTM4B −200∼+301 promoter region. The transcription start site depicted as right-point arrow. The relative scores predicted by JASPAR website online programs were registered under transcription factors name.

To explore the cis-acting element covering LAPTM4B promoter region, a 501 bp genomic DNA fragment (region from base 98787609 to 98788109 in the Homo sapiens chromosome 8 genomic contig) was analyzed. The LAPTM4B promoter region appears to be a G/C-rich promoter lacking canonical TATA box, CCAAT, and initiator elements. By computer analysis, potential binding sites for SP1, CREB1, Myf and Klf4 etc were found in the human LAPTM4B −200∼+301 promoter region (Fig. 1). The CREB1 gets the highest relative score among the transcription factors. After analyzing the LAPTM4B transcription initiation sites upstream and downstream of other seven species, we also found the CREB1 binding site in LAPTM4B promoter region in all the 7 vertebrate (Text S1).

### Luciferase Reporter Plasmids Identification

**Figure 2:**
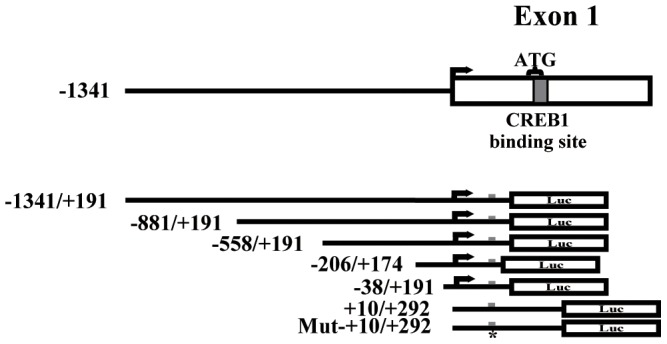
The upper panel is a mode chart of the 5′**-genomic structure of LAPTM4B gene.** Exon1 are depicted as a closed box, and 5′-region is black lines. The transcription start site depicted as right-point arrow. the gray box indicates the CREB1 binding site. The curly brace depicts the initiation codon in exon1. The lower panel shows the luciferase constructs ranging across the 5′-region. Closed boxes depict the luciferase. The CREB1 conserved binding site depicted as the gray box. Asterisk was referred to the mutated CREB1 binding site. (*P<0.05, *n* = 3).

In order to determine the authenticity of the plasmids, all the constructs were digested by restriction endonucleases Hind III and Xho I. The digested products were electrophoresis in 2% gel agarose. Fig. 2 is a mode chart of the 5′-genomic structure of LAPTM4B gene and luciferase constructs.

### Transcription Activity of the LAPTM4B Promoter Region

**Figure 3:**
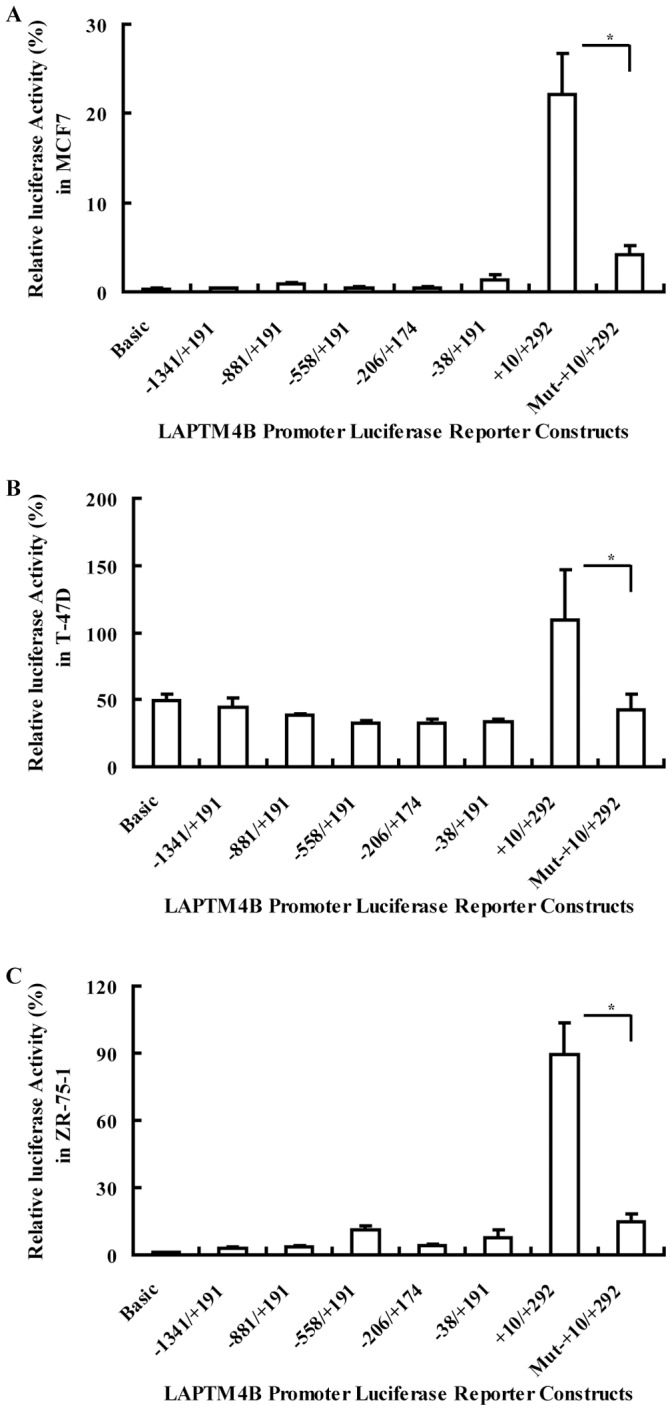
Relative luciferase activities from a series of 5′-deleted constructs and mutated construct. The luciferase mean values of three independence experiments performed in triplicates in MCF7, T-47D and ZR-75-1 respectively.

At 48h after transient transfection with various constructs, breast cancer cells were split for luciferase detection. The luciferase mean values of three independence experiments performed in triplicates were shown in Fig. 3. The transcriptional activity of the fragment upstream transcription start site (TSS) was found only slightly higher than negative control (pGL3-basic). In three breast cancer cell lines, fragment of +10∼+292 has the highest transcriptional activity among the six 5′-deleted constructs (Fig. 3). However, the basal transcriptional activities of fragment of +10∼+292 among the three cell lines are different, about 22%, 109% and 89% in MCF7, T-47D and ZR-75-1 respectively. The transcriptional activities of mutated constructs were significantly decreased than mutated before. The transcriptional activity decreased by approximately 80% in all the three breast cancer cell lines (Fig. 3).

### Analysis of the Ability of Transcription Factor CREB1 to Bind the LAPTM4B

**Figure 4:**
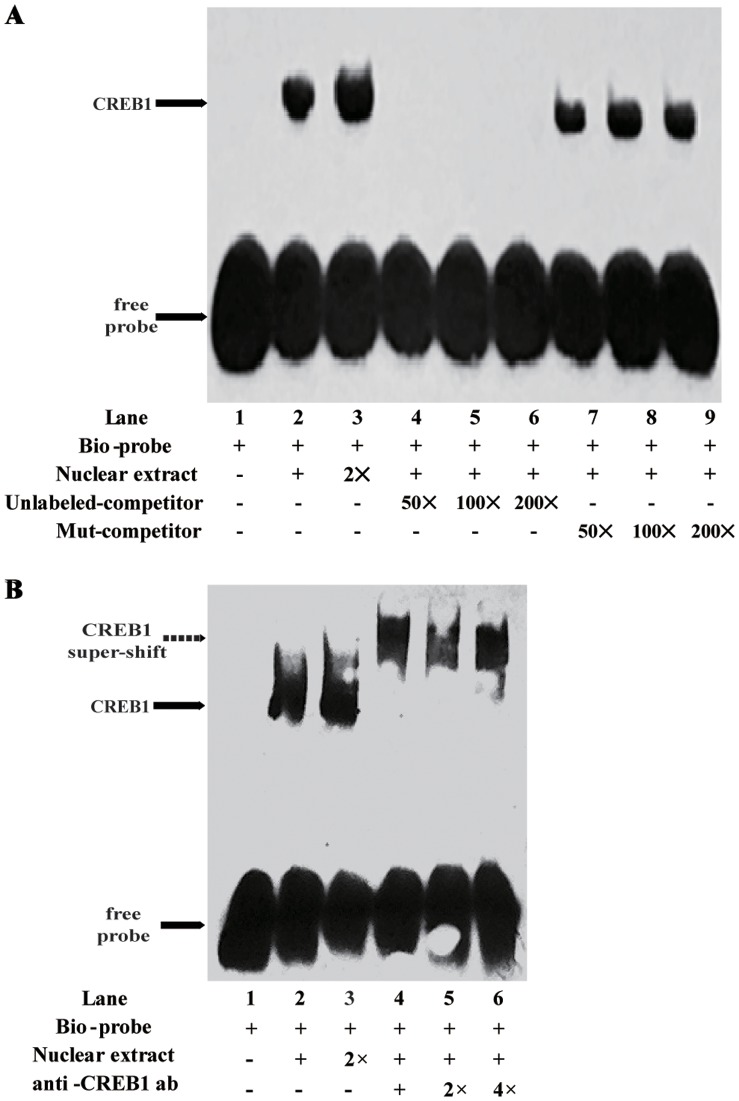
Analysis of the MCF7 nuclear extract proteins ability to bind dsDNA oligo targeting the LAPTM4B promoter. (A) EMSA. Nuclear extract proteins were incubated with biotin-labeled dsDNA oligo with excess of unlabeled competitors or CREB1 conserved binding site mutated competitors. (B) Super-shift. Nuclear extract proteins were incubated with biotin-labeled probe with different concentrations of CREB1 antibody. The antibody-CREB1-probe complex forms the supershit signal.

To determine whether CREB1 binds to the LAPTM4B core promoter, EMSA and super-shift assays were performed with MCF7 nuclear extract and biotin-labeled wild-type, unlabeled wild-type and mutant probes. After incubation with nuclear proteins, the 25 bp wild-type oligonucleotide containing the CREB1 binding motif formed protein-DNA complexes, but not with the mutant probe, which was competed out by the addition of unlabeled oligonucleotide probe. As showed in Fig. 4A, lane 1 was the migration of free-probe in the absence of nuclear extract and therefore no shift observed. Lane 2 was the CREB1-containing double-stranded DAN probe and showed a signal shift due to transcription factor binding (arrow). Lane 3 was bio-probe with 2-fold nuclear extract. The shift signal was stronger than lane 2. Lanes 4–6 showed that the shift signal can be inhibited from 50-fold, 100-fold, 200-fold excess unlabeled probe. Lanes 7–9 were CREB1 conserved binding site mutated competitors. The shift signal could not be inhibited from 50-fold, 100-fold, 200-fold excess mutated probe. Fig. 4B was the result from super-shift experiment. Lane 1 was the free probe. Lanes 2–3 were bio-probe with nuclear extract and 2-fold protein (arrow). Lanes 4–6 showed that the dsDNA-protein mixes with 1-fold, 2-fold and 4-fold specific anti-CREB1 antibodies had slower electrophoretic mobility (dashed arrow).

### Knockdown of the CREB1 Protein has a Negative Effect on LAPTM4B Transcription

**Figure 5:**
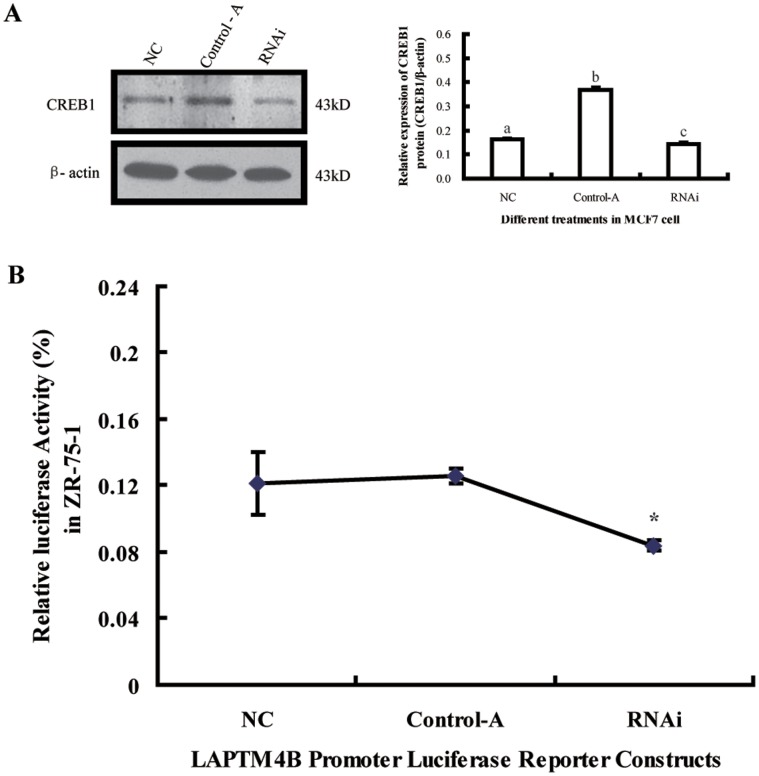
shRNA targeting of CREB1 specifically inhibits its expression and leads to impairment of LAPTM4B gene expression. (A) MCF7 cell were transfected with control shRNA (Conrol-A) or CREB1-specific shRNA (RNAi). NC represented the MCF7 cell without any treatment. Whole cell lysates were subjected to western blot analysis for CREB1 protein. The intensity of gray value of western blot bands. The different superscript letters represent significantly different groups (*p*<0.05). (B) Analysis of LAPTM4B gene transcript by real-time PCR after 48 h of shRNA transfection. (*P<0.05, *n* = 3).

Knockdown of CREB1 was performed by transient transfection of the shRNA targeting its mRNA. The CREB1 protein level in RNAi group decreased by approximately 50% compare with the control group: Control-A. However, the CREB1 protein level in Control-A group was higher than the NC group (Fig. 5A). Real-time PCR results showed that the level of LAPTM4B mRNA was significantly decreased in RNAi group compared with the group of Control-A (Fig. 5B). And the LAPTM4B mRNA in Control-A group was slightly increased compare with NC group.

## Discussion

In this study, a series of 5′-deleted constructs which generated from LAPTM4B promoter region were transiently transfected into breast cancer cell lines MCF7, T-47D and ZR-75-1 that expression LAPTM4B. By observing the intensity of luciferase (report gene), the transcriptional activity of different promoter regions were determined. Previous studies showed that in hepatoma carcinoma cell lines of BEL7402, HEPG2, HEL, the transcriptional activity of the fragment upstream TSS was found only slightly higher than empty vector (pGL3-basic) [13]. In this study (breast cancer cell lines MCF7, ZR75, T-47D), there was a similar trend for that observed in the HCC. The regulation of LAPTM4B promoter region upstream TSS is mainly negative. There may be two reasons for LAPTM4B negative regulation. First, due to its oncogene role, the expression of LAPTM4B should be strict controlled at a lower level in mature cells to preventing malignant transformation. We believed that there are trans-acting factors playing a negative regulatory role in LAPTM4B transcription. However, few references are available regarding LAPTM4B regulated by other transcription factors. As to this point, further research is needed. Second, the positive regulation region of LAPTM4B locates to TSS downstream. CREB1 binding sites were situated not only at 5′ ends but also in introns, exons, and TARs. Of the 215 CREB1 binding sites, 3% lay within a first exon [16]. So in the present study, the promoter region downstream TSS was paid attention to. A construct contained +10∼+292 promoter region was generated and transfected into breast cancer cell lines. The result of luciferase activity determining showed that this region was possessed a strong transcriptional activity. To our knowledge, the promoter region has not been identified previously. Analyzed by the IFTI-Mirage and JASPAR website online programs, the +10∼+292 fragment was found contained a CREB1 binding site. The CREB1 conservative binding sequence is an octamerci palindrome, that locate to +157∼+165. Fragments of −200∼+301 in the other 7 vertebrates (*Mus musculus*, *Rattus norvegicus, Danio rerio, Bos Taurus, Canis lupus familiaris, Gallus gallus, Pan troglodytes,)* were also analyzed. Among all the 7 vertebrates, CREB1 binding site was also found (supplement). Therefore, we believed that CREB1 is very important to LATM4B transcriptional regulation, and conserved in biological evolution.

CREB1 is a protein of 341 amino acids, which plays an important role in transcriptional regulation [22]. It has been studied in considerable detail in the nervous system but its role in the initiation/maintenance of cancer was only recently recognized [23]. Recent studies showed that CREB1 involved in the occurrence of breast cancer. CREB1 regulated FOXA1 expression as a critical downstream mediator of the EGFR-ErbB2 pathway [24]. CREB1 is a well-characterized ERK signaling transcription factor that is a down-stream target of active ERK through the mediation of the RSK and MSK family of kinases [14]; [22]; [25]. The down-regulation of the activity of ERK/STAT3/RI/PKA pathway, subsequently, leads to CREB1 phosphorylation reduced, followed by change in expression of CREB1-regulated genes, including Bcl2 [26]. Hsieh demonstrated that phthalates stimulated the aryl hydrocarbon receptor (AhR) and triggered the cyclic AMP (cAMP)/PKA/CREB1 pathway, which emanated from the phthalated-induced AhR promoted tumor genesis of ER-negative breast cancer [27].

We demonstrated that CREB1 could bind to the oligonucleotide probe generated from LAPTM4B promoter sequence through EMSA and super-shift experiments. In order to clarify the transcriptional regulation role that CREB1 played in LAPTM4B gene transcription, site-directed mutagenesis was introduced. Makhov [28] determined that mutation of CREB1 binding site resulted in loss of approximately 50% hZip1 promoter transcriptional activity. CREB1 also regulated TGF-β3 transcript and TGF-β3 promoter activity was decreased by 85% when the CRE site was mutated [29]. In this study, the mutated promoter led to the transcriptional activity decreased by approximately 80% in all the three breast cancer cell lines.

There were both positive and negative ways that CREB1 regulated target genes transcript. For example, CREB1 could inhibit MuSK [30] and c-fos [31] gene transcription. On the contrary, enforced expression of CREB1 shRNA constructs in MDA468 cells decreased STMN1 expression by >90% [32]. In this study, the levels of CREB1 and LAPTM4B in RNAi group were significantly decreased than Control-A group. It proved that CREB1 possessed the ability to positive regulate LAPTM4B transcription. The levels of CREB1 and LAPTM4B in Control-A group were higher than NC group. This may be because the exogenous DNA and transfection process induced cell injury. When cell faced to the survival pressure, the endogenous CREB1 and LAPTM4B was increased. When the intracellular protein level of CREB1 was knockdown by RNAi, the mRNA of LAPTM4B was declined simultaneously with the decrease of CREB1.

In conclusion, the regulation of LAPTM4B promoter region TSS upstream is mainly negative. The core promoter region of LAPTM4B gene was +10∼+292 fragment. CREB1 was an important factor regulating LAPTM4B gene transcription.

## Supporting Information

Text S1 **Analysis results of LAPTM4B promoter region in different species.** The nucleotide sequences are numbered with the putative transcription start site as +1. 501 bp (−200 ∼ +301) Genomic DNA fragment was predicted as LAPTM4B promoter region. Underlined letters indicate the upstream of the transcription start sites, and the potential CREB1 binding sites were indicated by the box. Italics letters in Homo sapiens refers to the 19 bp allele sequence.
(DOC)
