# Supplementary material for: cAMP Responsive Element Binding Protein-1 Is a Transcription Factor of Lysosomal-Associated Protein Transmembrane-4 beta in Human Breast Cancer Cells
Source: PLoS One. 2013 Feb 28;8(2):e57520. doi: 10.1371/journal.pone.0057520 (PMC3585361; doi:10.1371/journal.pone.0057520)
Supplement: Text S1 — Analysis results of LAPTM4B promoter region in different species. The nucleotide sequences are numbered with the putative transcription start site as +1. 501 bp (−200 ∼ +301) Genomic DNA fragment was predicted as LAPTM4B promoter region. Underlined letters indicate the upstream of the transcription start sites, and the potential CREB1 binding sites were indicated by the box. Italics letters in Homo sapiens refers to the 19 bp allele sequence. (DOC) [file pone.0057520.s001.doc]

**[*Homo sapiens*]：**

CGGGTGTGGTGGCAAGCGCCTGTAATCCCAGCTACTCGGTTGGTTGGGCAGGAGAATCGCTTGAACCCGGGAGGCGGAGGTTGCAGTGAGCCGAGATCGCGCCATTGCACTCCAGCCTGGGCGACAAGAGCGAGACTCTGTCTCAAAAAAAAAAAAAAAAGAAGTAGAAGGGAAGAAAATCGCAAGGAACTAGACTAAAAGAATCTCGACCCTTGAATGGAGTTACACGAACGGCCAGATGAAAGAAGGAAGGCCCGGACCTCCACTCAGGGCCGACTAGGGGACTGGCGGAGGGTGCACGCTGATGGATTTACTCACCGGGT*GCTTGGAGCTCCAGCAGCT*GGCTGGAGCCCGCGATGACGTCACGGACTCGGGTCACATGGCCAAGTCCGCCCCGCCCCCTCCCCGTCCCCGCCGCTGCAGCGGTCGCCTTCGGAGCGAAGGGTACCGACCCGGCAGAAGCTCGGAGCTCTCGGGGTATCGAGGAGGCAGGCCCGCGGG

**[*Mus musculus*]：**

CAGTTGACTGGAATAATCATTCTAAGCAGTTTTCCTTCCTTCCGCCAGTTTGCAAGATAACTACAATCCTAGCACAGCATTCACCGGTTATTTGCAAGCTAGATGTGAGGCAAACACAGCTGGAATGAATCTGAATCCTAACCAAAGGTGCACCCCATTTCCGGAGGAAAATTCTGGAGACTCCCTCAGGGTCCAGCAAGGAAGCATTTATTTGGCGCAGCGCTCCCAGCGCCCGCAGCTGGCTGGAGCCGGTGGTGACATCACGAGCCCGGGCAGGCCACGTGACCCACGGCTTCTCCTCCCCCCTGCCTCCTAGCGTCGCCGCGGTCTTCGAGCCCAGTGTCCCGTCGCAGCGGCCACTCGGAGCTCTCGGTACAGTGCAGAGCCAAGGGTCGGGCGAGCGGTCCGAGGGTCGGGGCGGCCAAGGAGAGGGGCAGGAGCAGCGGACCCAAGACGAAGCGAGCGACGCTCTCACAAACTTCCACGAGCGCCGGAGCTATG

**[*Rattus norvegicus*]：**

TGGCTGGAGCCCGCGGTGACATCACGAGCCCGGGCAGGCCACGTGACCCTCGGCCTCTCCTCCCCCGCCCCCTCGCGTCGCCGCGGTCCTCGAGCCCAGTGTCCCGCCGCAGCGGCCACTCGTAGCTCTCGGTGCAGTGCGGAGCCAAGGGTCGGGCGAGCGGTCGGAGCGTCGGGGCGCCAAGGAGAGGGGCTGGAGCAGCGGACCGTGTGAGCGGAGCCCGGGACGAAAAGAGAGAGACGGTCTCGCAAACTTGTGCGAGCGCCTGAGCTATGAAGATGGTCGCGCCCTGGACTCGGTTCTATTCCCACAGCTGCTGCCTGTGCTGCCATGTCCGCACCGGCACCATCCTGTTGGGCATCTGGTACCTGGTGAGCGCGGCCGGGCGTGGAGGGCGGCCCCCGGGGTCCCCACTCGCGCCGCTCACCCCGCACCGCTAAGTCGCTTAGTCTAGGTCGGCTTCGGTCTGGGGGCCGCCGGCGATCATCCGCCTAAAGTTGT

**[*Danio rerio*]：**

ACTTTCATTGTTCTGTGCGTAGACGTTCACTTTTAATTCAGCGTTTAGCGTTCAAAATGTTTGGAATATCGCTTTTTGGAATCGAGCGTGACCGGTCCGATTTGTTATAATATTCTCACGATATTTCTCAAGAAAATGAAGTAATTATCAGTGGATATTACTAAATGAAACCCAGAACAATGTTTGTCAGTAGGTAGAAAGTCTGTGAGTGTTTGTCTTTTGTCTTATTACACATTCTGTATTGGGTTGACACTGGCTTATGTCTCCTTTGTAAACAATAAAATATTCCAGTAATGCTTCATTATGGCTGGGCATATTTTTATACACACACACACACACACACACACACACACACACACACACACACGCTTGTACAGCTACACACACACACACACGCTTGTACAGCTATCTTCACGAGGGCTTCTCTTAGATGTAATGAACTGTGTATTTCCCCCCTACTTCATCGTCACGAGACACAATCTGCATTTTTACAGTTACAAA

**[*Bos taurus*]：**

TCTCGGTGGGGTGGGGTGGGGTGGGCTCCCCTAGTTTTCCCTCGGCGTTGGAACTACCCAACAACCCGGATGAATCTCGAGTCCGAGACGTCTGTTGTACGTGCTGAGCCCCAGAGTCGAAGACTTTCCAACGCTTTTCTCTTCACTCCCTCTTTCTCTTTTGAACGTGTATAATCAAGTCAAAATTAGACAGTGCTTTCCTTGGCCATTCCAATTTATAGAAGAAACCCTTAGGAGAACAGGAATGTTATTTGTGTAAGGAGTCCATATAAATACATAAATAAAACAAGAATTTCCTTAGACATTTACTTTATGATTTAAATTTGGTGACGGTTTTTGAGTTTGCTGGTCAAGGATTTTGACTGTGAGACCCTCTGATAATCCATGGCAAGGCAGACAGTCGGTGGCCGCCCTGCTGGCTGCTCTTGTCCGCGCGCGTCTGGGGTCACAGTGTGGCGTGTCGGGGGGACTGATGGATTTGGTCCTTTGTGCTGTCAAGTC

**[*Canis lupus familiaris*]：**

CAGCCAGCCCTCCTTACAGATGAGGACCCCATCCTCCTTACAGGTGAGGACCCCCGTCCTCCATACAGGTGAGGACCCCCATCCTCCATACAAATGAAGACCTCCATCCCCCTTACAAATGAGGACCCCGTCCTCCTTACAGATGAGGACCTCTGTCCTCCAGGCAGGGGCAAGGGACGTCTCCTCAAGACCCCCGGTCGATGGCCCACCAGCGCGTCCACCTAAGCCAATGCCGACCTAATACAGATTGGAATTCCTACAGGTTAGAGCTCTCAGAGAACTTGCACTCAAAAGGTGGAAAAAGTTGAGTGCACTCACCACGCGTGAAACCCTCCGGATAGGGTCCTGGAGGTCTCTCAGATCCCGGACGAGCCCCCAAATGTTGTGCCCAAGATTGCAAATCCGAGAAACCACCAAGGAGCCGACACGACACAGATGCAAACACACGAGGGTTTATTTATAAGCTGGAGCTTGGGTCCAAGAGCACCTGACAGAGCGGAG

**[*Gallus gallus*]：**

GTCGGGCTGGGTGTACGGAGCTCCTGGCAGAACCTTCTGCAGCATTTCCCGGAGAGAACGACACCGCTTCCTCCAAGGAGCCCCCCGGGGCTGCGATGACCGCTCCGGGACCGCCTCAGCCCCCTCCCTCCGCCGCCGCCGCCGCCGCCTCGAGTCCCGCCCGCCGGGAGGGGCGGTCCCGGCCCGGCCCAACACCGCCCCCGCGGAGCAGCGCGGCGAGAGGCGCGGAGCGCGGAACCCCGAGAGCCGCGGAGATGCTGAGCTCGCCCTGGGCGCGCTTCTATTCCAACAGCTGCTGCCTCTGCTGCCATGTCCGCACCGGCACCATCATTCTCGGCGTCTGGTACCTGGTAAGCGGCCCCGCCGTGCCCGCGTCCCCCCGGGGCGGGGGGATTCTTCGGTCTCGTGGGCCGGCACGGTTGCGCTCCGCCAGCTGTGTCTGGGGGGGGAAAGCCCTCTCCCCGTTACCAGCATTATGGCGTCGGGTGAGCTCCTGTGCTT

**[*Pan troglodytes*]：**

GCGTGGTGGCGGGCGCCTGTAGTCCCAGCTACTAGGGAGGCTGAGGCAGGAGAATGGCGTGAACCCGGGAGGTGGAGCTTGTAGTGAGCCGAGATCGCACCACTGCAATCCAGCCTGGGTGACAGAGCAAGACTCTGTCTCAAAAAAAAAAAAAAAAAAAAAAAGCTACCGGAAGCACAGTGAGGATGTCCTTGACACACATCCTATTTTCTGGGAAAAGATTACTACCACAGTAATTGAGCTGTGAAGCGGAGACAAATTGCTCTCGGTGGTGGTTCAAAGTACTGCAATTGACTGGAATAGCACCGCGCAGTTTTCCTTCCTCTCGAGCAAGATAAGAGTGATAGGAGCTGTATCGATTACCTGCAAGATAGAAGTAGAAGCGGGCCGGGTGCGGTGGCTCACGCCTGTAATCACAGCACTTTGGGAGGCTGAGGCGGGTGGATCATTCGACGTCAGGAGTTCCAGACCAGCCTGACCAACATGGTGAAACCCCGTCTC
